# Amivantamab for the treatment of *EGFR* exon 20 insertion mutant non-small cell lung cancer

**DOI:** 10.1080/14737140.2022.2016397

**Published:** 2021-12-28

**Authors:** Simon Vyse, Paul H Huang

**Affiliations:** Division of Molecular Pathology, The Institute of Cancer Research, Sutton, UK

**Keywords:** Amivantamab, EGFR, exon 20 insertions, lung cancer, monoclonal antibody

## Abstract

**Introduction:**

Amivantamab is a monoclonal bispecific anti-EGFR-MET antibody that is the first targeted therapy to be approved for non-small cell lung cancer (NSCLC) patients harboring *EGFR* exon 20 insertion mutations following progression on chemotherapy, marking a watershed moment for a class of mutations which is generally associated with poor outcomes.

**Areas covered:**

In this article, we outline the drug profile of amivantamab compared with EGFR kinase inhibitors under evaluation in *EGFR* exon 20 insertion mutant NSCLC. We also review the efficacy and safety data reported from the CHRYSALIS phase I trial, which forms the basis of the recent approval of amivantamab.

**Expert opinion:**

Unlike small molecule EGFR kinase inhibitors, amivantamab has an extracellular mode of action and dual activity against EGFR and MET. It remains to be determined what role MET inhibition plays in toxicity and efficacy and whether dual target inhibition can delay the onset of drug resistance in these cancers. Due to its large molecular size, amivantamab is expected to have poor activity to treat brain metastases. Building on the clinical data so far, future trials that will evaluate combination treatments with brain-penetrant EGFR kinase inhibitors will be critical to move the drug toward a first-line treatment.

## 1. Introduction

In the last two decades, advances in the molecular characterization of non-small cell lung cancer (NSCLC) have resulted in improvements in survival outcomes for selected groups of patients. The ability to profile tumors beyond histology and identify underlying genetic oncogenic drivers has led to an era of precision oncology and the effective use of targeted therapies in patients with anaplastic lymphoma kinase (*ALK*) translocations [1], *ROS1* proto-oncogene receptor tyrosine kinase (*ROS1*) rearrangements [2], neurotrophic receptor tyrosine kinase (*NTRK*) gene fusions [3], B-raf proto-oncogene, serine/threonine kinase (*BRAF*) mutations [4], *RET* proto-oncogene (*RET*) gene fusions [5], *MET* exon 14 alterations [6], Kirsten rat sarcoma virus (*KRAS*) mutations [7] and epidermal growth factor receptor (*EGFR*) mutations [8,9].

The successful use of the first-generation EGFR kinase inhibitors gefitinib and erlotinib to treat *EGFR* mutant positive NSCLC in the early 2000s were among the first of these developments to pave the way for targeted therapies in lung cancer [10,11]. Activating mutations in *EGFR* are one of the most prevalent oncogenic drivers in NSCLC accounting for 15–20% of adenocarcinoma patients in Caucasian populations, with an increased prevalence of up to 50% of patients in Asian populations [12–15]. From clinical experience, it is now clear however that not all *EGFR* mutations in NSCLC are associated with sensitivity to EGFR kinase inhibitors. In-frame base pair insertions in exon 20 result in constitutive activation of EGFR, but unlike the more common ‘classical’ activating *EGFR* mutations (L858R and exon 19 deletions), they have been associated with *de novo* resistance to targeted EGFR kinase inhibitors [16–18] *EGFR* exon 20 insertions vary in length (between 3–21 base pairs) and point of insertion (between codons 767 and 774) but when collectively grouped together, this class of mutations are the third most common type of *EGFR* mutation in NSCLC reported at between 4–10% of all *EGFR* mutations [17,19–21]. The epidemiology of *EGFR* exon 20 insertions matches the characteristics seen in classical *EGFR* mutant NSCLC; they are more common in Asian, female, never-smoker patients [17,20].

The structural features of EGFR exon 20 insertion kinases and their differences compared to classical EGFR mutants form the basis of their insensitivity to EGFR kinase inhibitors [19]. Classical *EGFR* mutations result in constituently active EGFR activity but importantly, the mutant receptors have greatly reduced affinity for ATP compared to wild-type (WT) EGFR [22,23]. The result is that inhibitors such as gefitinib and erlotinib, which compete with ATP for binding in the catalytic pocket of EGFR, are therefore much more likely to bind to mutant versus WT EGFR with relatively greater affinity due to alleviation of the competitive pressure with ATP. WT EGFR inhibition is associated with toxicities in patients, primarily rash and diarrhea [24,25]. Good mutant selectivity is therefore what affords EGFR kinase inhibitors such a wide therapeutic window in classical *EGFR* mutant NSCLC, allowing treatment with high enough doses that can be clinically effective while maintaining a tolerable safety profile for the patient. EGFR exon 20 insertions activate EGFR kinase activity without significantly impairing the ATP affinity of the receptor compared with WT EGFR [19]. Moreover, compared with classical EGFR mutants, EGFR exon 20 insertion mutant kinases harbor a more compact drug-binding site imposed by a more rigid conformation of a structural feature of EGFR known as the C-helix [26]. Together, these features mean that kinase inhibitors such as gefitinib and erlotinib lose their mutant selectivity altogether, and will target EGFR exon 20 insertions and WT EGFR with similar potency. This results in an extremely small therapeutic window to treat *EGFR* exon 20 insertion mutant patients with kinase inhibitors that have been approved for classical EGFR mutants. Achieving a dose which is both clinically effective and tolerable to patients without generating significant toxicity associated with WT EGFR inhibition has therefore been a major challenge for the treatment of *EGFR* exon 20 insertion NSCLC to date.

EGFR kinase inhibitors that are approved for NSCLC with *EGFR* L858R mutations or exon 19 deletions are ineffective in the vast majority of *EGFR* exon 20 insertion mutant NSCLC patients. From retrospective studies, first-generation kinase inhibitors gefitinib and erlotinib typically have very low response rates (RR) reported (8–27%) and less than 3 months median progression-free survival (PFS) in patients with *EGFR* exon 20 insertions [27,28]. The exceptions to the rule are patients which harbor A763_Y764insFQEA insertions; patients which harbor these mutations have shown partial responses to first-generation EGFR kinase inhibitors in-line with classical EGFR mutations [17,29]. Underlying this sensitivity is the observation that A763_Y764insFQEA insertions and other insertions which occur directly within the C-helix of EGFR may share a mechanism of activation and structural similarity resembling the L858R mutant kinase [19]. However, insertions at this location are relatively rare; over 90% of *EGFR* exon 20 insertions in NSCLC occur in the region following the C-helix and have been associated with EGFR kinase inhibitor resistance [16].

Second-generation EGFR kinase inhibitors including neratinib and afatinib are similarly ineffective in patients with *EGFR* exon 20 insertions, with <3 months median PFS [30,31]. The third-generation EGFR kinase inhibitor osimertinib, originally designed to overcome drug resistance caused by the secondary *EGFR* T790M mutation, has become favored as a first-line therapy in the context of classical *EGFR* mutant NSCLC based on impressive median PFS of >18 months [32]. Although initial pre-clinical data [26,33,34] and clinical case reports of response to osimertinib [35,36] were promising, the use of osimertinib in the treatment of *EGFR* exon 20 insertion patients is still not clearly defined. The ongoing phase II clinical trial ECOG-ACRIN EA5162 (NCT03191149) to prospectively evaluate osimertinib for patients with *EGFR* exon 20 insertion NSCLC failed to meet its primary endpoint of 30% RR, although preliminary results from 17 evaluable patients demonstrated a RR of 24% and median PFS of 9.6 months [37,38]. In contrast, another phase II trial to study osimertinib in *EGFR* exon 20 insertion NSCLC (LU17-19) reported a 0% RR and median PFS of 3.5 months in 15 *EGFR* exon 20 insertion patients [39]. Based on current data, it looks unlikely that osimertinib will be universally effective for *EGFR* exon 20 insertion patients but may have limited clinical utility in a subset of patients. Ongoing and future trials to optimize the dosing regimen of osimertinib including a high dose regimen (160 mg daily versus the standard 80 mg daily dose) may help to further improve treatment benefit in this patient population [37,38]. However, in the context of the recent emerging landscape of targeted agents under investigation or now approved for *EGFR* exon 20 insertion NSCLC, it is likely that off-label osimertinib use will be limited to patients without access to clinical trials or approved standard-of-care therapies.

Due to the lack of clinical activity of approved EGFR kinase inhibitors for *EGFR* exon 20 insertions, the standard of care for the majority of patients has remained cytotoxic chemotherapy comprising a platinum based agent such as cisplatin or carboplatin combined with a taxane or pemetrexed in the first line setting [40–42]. As expected, the clinical outcomes of *EGFR* exon 20 insertion patients are considerably worse than NSCLC patients with classical *EGFR* mutations treated with a targeted EGFR inhibitor. For example, recent real-world data identified an 11.8% RR, median PFS of 8.9 months and overall survival (OS) of 29.3 months in 17 *EGFR* exon 20 insertion patients treated with platinum-doublet chemotherapy, compared to 57.9% RR, median PFS of 13.6 months and OS of 43.4 months for patients with classical *EGFR* mutations who received an EGFR inhibitor therapy [43].

In order to address this clinical unmet need, several therapeutics with the potential to target the exon 20 insertion EGFR mutant receptor directly have been assessed in the clinic. In 2021, two targeted therapies have been approved for *EGFR* exon 20 insertion mutant NSCLC: the small molecule EGFR kinase inhibitor, mobocertinib, and the anti-EGFR-MET bispecific antibody, amivantamab [44,45].

## 2. Overview of the market: the landscape of *EGFR* exon 20 insertion specific therapies in NSCLC

There are a number of agents currently under evaluation to treat NSCLC harboring *EGFR* exon 20 insertions at various stages of clinical development (Table 1). Broadly, these include 2 classes of drug: small molecule kinase inhibitors that can inhibit the EGFR exon 20 insertion mutant kinase activity, or monoclonal antibodies (mAbs) that bind to EGFR extracellularly and promote receptor internalization and degradation and blockade of receptor signaling. In the classical mutant *EGFR* NSCLC setting, kinase inhibitors remain the gold standard of treatment due to good mutant selectivity [22,23] and capacity to cross the blood-brain barrier to treat brain metastases [32] and combination treatments with mAbs are yet to improve patient outcomes [46]. The unique mechanisms of action of mAbs may however present an opportunity in *EGFR* exon 20 insertion mutant NSCLC where the therapeutic window for kinase inhibitors is greatly reduced. Currently under evaluation in *EGFR* exon 20 insertion mutant NSCLC are small molecule kinase inhibitors (poziotinib, mobocertinib, CLN-081, BDTX-189, DZD9008) as well combination treatments of standard EGFR kinase inhibitors approved for classical *EGFR* mutant NSCLC (afatinib or osimertinib) with anti-EGFR mAbs (cetuximab, necitumumab and JMT101). Lastly the mAb amivantamab which targets both EGFR and MET receptors is being evaluated as a monotherapy or in combination with chemotherapy or EGFR kinase inhibitors – these studies will be discussed in greater detail in subsequent sections.

**Table 1.** Summary of efficacy data for agents undergoing clinical evaluation for EGFR exon 20 insertion mutant NSCLC. TKI: tyrosine kinase inhibitor; mAb: monoclonal antibody; EGFR: epidermal growth factor; NSCLC: non-small cell lung cancer; RR: response rate; PFS: progression-free survival; NR: not reported

Name of agent	Type of agent	Trial phase	Clinical Trial	Patient population (n)	RR (%)	Median PFS (months)	Reference
TKIs							
Poziotinib	EGFR TKI	Phase II	ZENITH20 NCT03318939	Treatment-naïve *EGFR* exon 20 insertion mutant NSCLC (n = 79)	27.8%	7.2	[53]
Mobocertinib	EGFR TKI	Phase I/II	EXCLAIM NCT02716116	*EGFR* exon 20 insertion mutant NSCLC with prior platinum-based chemotherapy (n = 114)	28%	7.3	[47,56]
CLN-081	EGFR TKI	Phase I/IIa	NCT04036682	*EGFR* exon 20 insertion mutant NSCLC with prior platinum-based chemotherapy (n = 25)	40% (interim data, unconfirmed)	NR	[60]
BDTX-189	EGFR TKI	Phase I/II	MasterKey-01 NCT04209465	Relapsed advanced solid tumors with EGFR/HER2 alterations and no available standard therapy (n = 27)	7% (interim data, unconfirmed)	NR	[61]
DZD9008	EGFR TKI	Phase I/II	NCT03974022, CTR20192097	*EGFR* or *HER2* exon 20 insertion mutant NSCLC (n = 31)	48.4% (interim data)	NR	[62]
mAbs							
Amivantamab	anti-EGFR-MET mAb	Phase I	CHRYSALIS NCT02609776	*EGFR* exon 20 insertion mutant NSCLC with prior platinum-based chemotherapy (n = 81)	40%	8.3	[63]
mAb + TKI combinations							
Afatinib + Cetuximab	EGFR TKI + anti-EGFR mAb	Phase II	AFACET NCT03727724	*EGFR* exon 20 insertion mutant NSCLC (n = 17)	47% (interim data)	5.5	[67]
Osimertinib + Necitumumab	EGFR TKI + anti-EGFR mAb	Phase I	NCT02496663	*EGFR* mutant NSCLC	50% (2/4 *EGFR* exon 20 insertion pts)	5.3	[68]
Afatinib or osimertinib + JMT-101	EGFR TKI + anti-EGFR mAb	Phase Ib	NCT04448379	*EGFR* exon 20 insertion mutant NSCLC	NR	NR	[48]

### 2.1. Poziotinib

Poziotinib (formerly HM781-36B) is an irreversible, covalent kinase inhibitor that targets EGFR and HER2 and was among the first small molecule to demonstrate activity against *EGFR* exon 20 insertion mutations [26,49]. Having initially shown limited efficacy against NSCLC patients with classical *EGFR* mutations who had acquired the T790M resistance mutation [50], subsequent *in silico* modeling highlighted a potential activity for poziotinib to target the structure of EGFR exon 20 insertions [26]. 3D modeling was used to predict that the flexible structure of poziotinib would allow the drug to tightly bind the compact EGFR exon 20 insertion binding pocket, which was supported by evidence of inhibition *in vitro* in cell line and patient-derived xenograft models. Although early clinical data were promising [51], recent data from the phase II ZENITH20 trial (NCT03318939) failed to meet its primary endpoint. In a cohort of 115 *EGFR* exon 20 insertion NSCLC patients (median of two prior lines of therapy) only a limited RR of 14.8% and median PFS of 4.2 months was reported [52]. In a separate cohort of 79 patients within the same trial with no prior lines of therapy showed a RR of 27.8% and median PFS of 7.2 months [53]. The ZENITH20 trial highlighted toxicity concerns due to WT EGFR inhibition by poziotinib, with 63% of patients showing grade 3 or 4 treatment related adverse events (TRAEs) and 68% requiring dose reductions from the initial 16 mg once daily dose. The ZENITH20 trial is ongoing and additional cohorts will explore whether a split dosing strategy of 8 mg twice daily poziotinib can reduce toxicity without compromising therapeutic efficacy.

### 2.2. Mobocertinib (TAK-788)

Mobocertinib (formerly known as TAK-788/AP32788) is an irreversible, covalent kinase inhibitor of EGFR and HER2 with activity against EGFR exon 20 insertion mutations [54]. A phase I/2 trial (EXCLAIM; NCT02716116) which evaluated 160 mg mobocertinib once daily in 114 *EGFR* exon 20 insertion patients with prior platinum-based therapy reported a RR of 28% and median PFS of 7.3 months which is superior to poziotinib [44,55]. Based on these results, the Food and Drug Administration (FDA) granted an accelerated approval for mobocertinib as a second-line therapy to treat NSCLC with *EGFR* exon 20 insertion mutations with progression on platinum-based chemotherapy in September 2021 [44]. Mobocertinib has a more tolerable toxicity profile compared to poziotinib, with grade 3 or 4 TRAEs occurring in 40% of patients in the dose escalation cohort of 136 patients, the most common TRAEs being diarrhea (83%), nausea (43%) and rash (33%) [56]. The phase III EXCLAIM-2 cohort (NCT04129502) will directly compare first-line platinum-based chemotherapy versus mobocertinib in treatment-naïve *EGFR* exon 20 insertion NSCLC patients, with median PFS as a primary endpoint [57]. This study will be the first to compare a novel therapy with activity against EGFR exon 20 insertions as a first-line treatment against standard-of-care chemotherapy. Importantly, EXCLAIM-2 will include patient stratification for the presence of brain metastases. Mobocertinib was previously reported to have a lower RR in patients with baseline brain metastases compared to patients without (25% vs 56%) [56], however, the results from the EXCLAIM-2 trial will provide more robust evidence to help clearly establish the CNS activity of mobocertinib.

### 2.3. CLN-081 (TAS6417)

CLN-081 (formerly known as TAS6417/TPC-064) is an irreversible, covalent kinase inhibitor which was specifically designed to target the ATP-binding site of the EGFR exon 20 insertion kinase domain and has shown good mutant versus WT EGFR selectivity in pre-clinical data [58,59]. This inhibitor is at an early stage in clinical development, with a phase I/IIa trial (NCT04036682) currently ongoing. Interim data from this trial in 25 evaluable patients has reported an unconfirmed RR of 40% [60].

### 2.4. BDTX-189

BDTX-189 is a novel irreversible kinase inhibitor designed to inhibit a broad range of ‘undrugged’ oncogenic mutations of both EGFR and HER2 kinases including extracellular domain, allosteric mutations of *HER2* in addition to *EGFR* and *HER2* exon 20 insertion mutations [61]. Based on pre-clinical data showing mutant versus WT selectivity across 48 allosteric HER2 mutant variants and *EGFR or HER2* exon 20 insertion mutants, as well as tumor growth inhibition *in vivo*, a phase I/II trial, MasterKey-01 (NCT04209465), is ongoing to determine the dosing regimen and recommended phase II dose (RP2D) of BDTX-189 monotherapy [61]. This pan-cancer trial cohort includes patients with locally advanced or metastatic solid tumors with *EGFR* or *HER2* exon 20 insertion mutations, in addition to allosteric *HER2/HER3* mutations, *HER2* amplification, or *EGFR* exon 19 deletion or L858R mutation. In this cohort, preliminary data has been reported with 7% unconfirmed RR in 27 evaluable patients, however further data will be required to determine the efficacy for the *EGFR* exon 20 insertion NSCLC subgroup.

### 2.5. DZD9008

Two phase I/II studies (CTR20192097 and NCT03974022) are ongoing to assess another irreversible, small molecule kinase inhibitor rationally designed to target *EGFR* exon 20 insertion mutants, DZD9008 [62]. These trials are evaluating DZD9008 in NSCLC with *EGFR* or *HER2* mutations, and a pooled analysis was used to determine RP2D of 300 mg once daily. At this dose, a RR of 48.4% was observed in 31 evaluable patients, across multiple different types of *EGFR* exon 20 insertion mutations.

### 2.6. Antibody and EGFR kinase inhibitor combinations

In addition to monotherapy with small molecule kinase inhibitors, there is biological rationale to evaluate treatments of kinase inhibitors in combination with mAbs that target the extracellular domain of EGFR in *EGFR* exon 20 insertion mutant NSCLC. *In silico* structural modeling of two *EGFR* exon 20 insertions, D770_P772del_insKG and D770> GY, were predicted to favor the formation of EGFR dimers [64]. Cetuximab, an antibody which binds the extracellular domain of EGFR and blocks dimer formation [65], has therefore been explored as a potential treatment in combination with EGFR kinase inhibition in *EGFR* exon 20 insertion mutant NSCLC. This hypothesis is supported by a clinical study that identified partial responses to treatment with a combination of cetuximab and the second-generation EGFR kinase inhibitor, afatinib, in three out of four *EGFR* exon 20 insertion positive patients with a median PFS of 5.4 months [80]. Case reports from a phase I trial evaluating a combination of cetuximab and erlotinib also identified a remarkable 3.5 years PFS for one NSCLC patient harboring a D770> GY exon 20 insertion, highlighting the possibility of durable responses to mAb and EGFR kinase inhibitors for certain patients with *EGFR* exon 20 insertions [64,66]. The phase II trial AFACET is ongoing to assess the combination of afatinib and cetuximab in *EGFR* exon 20 insertion NSCLC patients (NCT03727724) [67]. Interim data reported from the first 17 patients enrolled in the study identified a RR of 47% and median PFS of 5.5 months. A potentially challenging toxicity profile was highlighted by this study, with 59% of patients experiencing grade ≥3 or 4 TRAEs and 82% of patients requiring dose reduction.

Other ongoing studies assessing antibody and kinase inhibitor combinations include a phase I study evaluating necitumumab, an anti-EGFR mAb, with osimertinib (NCT02496663), a combination which has shown response in 2 out of 4 patients with *EGFR* exon 20 insertion mutations [68]. JMT-101 is an anti-EGFR mAb which has shown favorable a toxicity profile in patients with colorectal cancer [81] and is being assessed in combination with afatinib or osimertinib in a phase Ib trial in patients with *EGFR* exon 20 insertion mutations (NCT04448379).

## 3. Introduction to amivantamab

### 3.1. Discovery and biochemistry of amivantamab

Amivantamab (formerly JNJ-61186372) is a human monoclonal, IgG1-based bispecific antibody that targets EGFR and MET and was developed by Janssen Biotech in collaboration with Genmab using Genmab’s DuoBody technology platform [69]. Both EGFR and MET pathways have been implicated in driving tumor growth in lung cancer [11,70–72] and in particular, MET pathway activation via upregulation of MET expression has frequently been described a mechanism of EGFR kinase inhibitor resistance [73,74]. Based on this knowledge, Neijssen *et al*. sought a therapeutic strategy for NSCLC by dual targeting of both pathways with a bispecific antibody [69]. The authors employed a series of empirical screening phases to identify the optimal EGFR and MET monovalent antibodies that could achieve maximum inhibition of their respective pathways, without triggering the undesired agonist effect of reception dimerization and activation, which has previously been observed for some MET bivalent antibodies [75]. Using a controlled antigen-binding fragment (Fab)-arm exchange (cFAE) platform, a panel of 40 bispecific EGFR-MET-antibodies were generated from 5 MET parental mAbs x 8 EGFR parental mAbs, each with distinct combinations of Fab domains, the region of the antibody which binds to its target antigens [69]. These antibodies were screened over 4 selection phases: 1. A binding assay, with the bispecific antibody required to bind both targets with half maximal effective concentration (EC_50_) < 1 µg/ml in monovalent format, 2. A MET phosphorylation assay, to confirm that bispecific antibodies did not induce MET phosphorylation in an unstimulated lung cancer cell line, A549, and therefore did not have unintentional agonist activity, 3. Proliferation assays, to confirm the ability of bispecific antibodies to inhibit cell proliferation of the pancreatic cell line KP4, driven by a hepatocyte growth factor (HGF)-MET autocrine loop, and the H1975 lung cancer cell line, driven by a double mutant *EGFR* (L858R and T790M) and 4. An EGFR phosphorylation assay, to eliminate bispecific antibodies that induced EGFR phosphorylation in the absence of EGFR ligand in A549 cells. Following this screening process, the antibody with the most optimal properties was selected and grown in a proprietary cell line to generate a mAb with a low fucose fragment crystallizable (Fc) form to enhance antibody-dependent cellular cytotoxicity (ADCC), resulting in the final molecule amivantamab.

The crystal structure of the anti-MET Fab of amivantamab bound to MET was solved by Neijssen *et al*. to better understand the mechanism of MET inhibition [69]. Amivantamab binds to a particular extracellular region of MET known as the Sema domain, which is required for HGF-induced receptor dimerization and activation [76]. The large interface of the Fab domain of amivantamab with the Sema domain of MET blocks binding of the β-chain of HGF to MET and therefore prevents ligand-induced activation. Mapping of the EGFR epitope bound by amivantamab was performed by a combination of site-directed mutagenesis and flow cytometry binding assays to identify residues K443, K465, I467, and S468 in the extracellular EGFR domain III, which shows partial overlap with the epitope bound by the mAb cetuximab [76].

### 3.2. Pharmacodynamics

**Figure 1.**
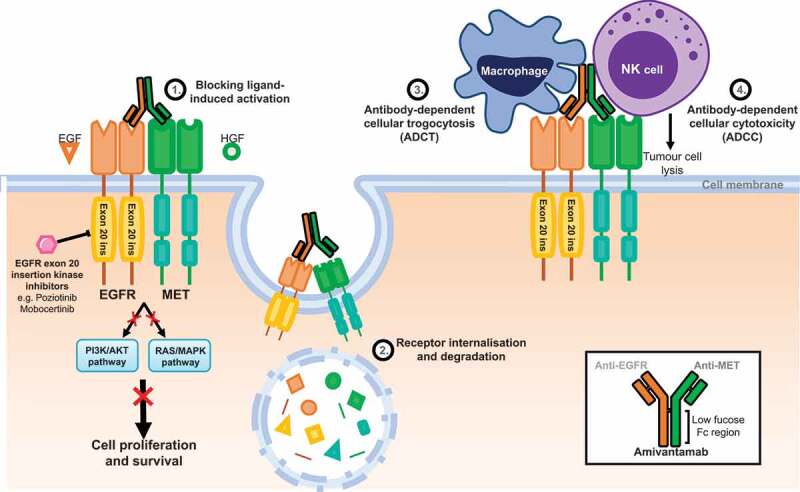
Mechanisms of action of amivantamab to target NSCLC cells. Small molecule kinase inhibitors e.g. poziotinib and mobocertinib bind to the kinase domain of *EGFR* exon 20 insertion mutants and block downstream survival signaling. In contrast, the anti-EGFR-MET antibody amivantamab has been described to have 4 mechanisms to block EGFR/MET signaling: 1. Extracellular binding of amivantamab to receptors prevents further activation by blocking binding of ligands, 2. Antibody-bound receptors are internalized and degraded, 3. Macrophage/monocyte recruitment triggers antibody-dependent cellular trogocytosis (ADCT), from the Greek *trogo* ‘to gnaw’ – a process that results in transfer of small membrane fragments including EGFR/MET receptors from tumor cells to lymphocytes and 4. Activated NK cells directly lyse tumor cells via antibody-dependent cellular cytoxicity (ADCC)[78–80].

Using purified extracellular domains (ECD) of EGFR and MET, the affinity (K_d_) of amivantamab was determined to be 1.4 nmol/L for EGFR-ECD and 40 pmol/L for MET-ECD using *in vitro* competitive binding assays, and amivantamab was capable of binding both EGFR-ECD and MET-ECD simultaneously [77]. Using *in vitro* and *in vivo* models, amivantamab was demonstrated to have four distinct mechanisms of action to inhibit cell proliferation (Figure 1) including blockade of receptor activation, internalization and receptor degradation in lysosomal compartments as well as trogocytosis, and induction of ADCC. Firstly, ligand-induced receptor activation is blocked by amivantamab. Data from a panel of *in vitro* cell line models that harbor *EGFR* WT, *EGFR* exon 19 deletion or L858R alone or in combination with T790M, and both amplified and non-amplified WT *MET* showed that amivantamab inhibited both EGF-induced phosphorylation of EGFR and HGF-induced phosphorylation of MET in a dose-dependent manner with half maximal inhibitory concentration (IC_50_) < 100 nmol/L in all models [77]. Amivantamab treatment results in receptor expression downmodulation, which was shown *in vivo*. Mice were implanted with the H1975 cell line engineered to express HGF (H1975-HGF) in order to induce activation of both EGFR and MET pathways. Following treatment with 1, 5 and 20 mg/kg amivantamab, Western blot analysis of tumors from these mice confirmed both total and phosphorylated EGFR and MET receptor expression was significantly reduced at all doses compared with vehicle-treated mice. This internalization is mediated by two mechanisms: 1. Internalization of both EGFR and MET receptors in tumor cells and degradation in lysosomal compartments [78] and 2. trogocytosis (named antibody-dependent cellular trogocytosis, ADCT) [79]. This latter mechanism of ADCT involves an Fc-dependent interaction of the antibody with the Fcγ receptor on monocytes and macrophages, resulting in antibody-mediated transfer of membrane fragments from tumor cells to immune effector cells thereby downmodulating the receptor in tumor cells. Lastly, by observing tumor cell lysis of the lung cancer lines H292 (*EGFR* WT) and H1975 (*EGFR* L858R/T790M) treated with amivantamab in the presence of human peripheral blood mononuclear cells (PBMCs) *in vitro*, amivantamab was demonstrated to induce ADCC in a similar fashion to cetuximab [77].

The effect of amivantamab on EGFR exon 20 insertion mutants specifically was first described by Yun *et al*. [78]. Using Ba/F3 cells expressing five distinct EGFR exon 20 insertion mutants, cell viability was assessed following treatment with amivantamab ranging from 0.05 to 1 mg/ml. Cell viability was significantly reduced in all five models when compared with minimal antiproliferative effects following treatment with gefitinib or osimertinib. The cell viability assay was also used to compare mutant versus WT EGFR selectivity between amivantamab and poziotinib treatment in the Ba/F3 cells. Amivantamab demonstrated better mutant selectivity when compared to poziotinib, with particularly potent effects on three insertions (V769_D770insASV, D770delinsGY, H773_V774insH, IC_30_ 0.1–0.6 mg/ml). Notably however, similar to poziotinib, amivantamab demonstrated potent inhibition of cell viability of Ba/F3 cells expressing WT EGFR (IC_30_ 0.9 mg/ml) and was not selective for two insertions tested (Y764_V765insHH and D770_N771insSVD, IC_30_ 1.5 mg/ml and 1.4 mg/ml respectively). These results highlight a potential for heterogeneous responses and toxicity profiles across patients with distinct *EGFR* exon 20 insertions.

### 3.3. Pharmacokinetics and metabolism

NSCLC patients with *EGFR* exon 20 insertion mutations that were enrolled into the phase I CHRYSALIS study (NCT02609776) were treated with amivantamab intravenously once weekly for the first cycle (28 days) and once every 2 weeks for subsequent 28-day cycles, starting at week 5 [63]. Based on serum concentration data after treatment on cycle 2, day 1, amivantamab was observed to have linear pharmacokinetics at 350 mg up to the maximum assessed dose of 1,750 mg, with non-linear pharmacokinetics below 350 mg. Mean nonspecific linear clearance of amivantamab was 0.36 L/d with a mean half-life of 11.3 days. By measuring the circulating serum concentrations of free, unbound MET and EGFR, saturation of targets begun at 350 mg amivantamab for EGFR and 140 mg amivantamab for MET after a single dose, which was consistent with onset of associated on-target toxicities of rash for EGFR and hypoalbuminemia and peripheral edema for MET. Complete saturation of both targets for the duration of the dosing period was achieved at ≥ 700 mg of drug. The investigators established a two-tiered RP2D of 1,400 mg for patients ≥ 80 kg and 1,050 mg for patients < 80 kg and demonstrated similar exposure by measuring pharmacokinetic properties including area under the concentration-time curve following amivantamab infusion at steady state during a dosing interval of 14 days and steady state concentration at the end of infusion. To assess the impact of drug immunogenicity, the authors determined a very low incidence of anti-amivantamab antibodies following treatment (1% of evaluable patients across all doses) with no observable impact on clinical activity or safety.

## 4. Clinical efficacy of amivantamab

### 4.1. Phase I and Phase II

The phase I and II trials which are ongoing to evaluate amivantamab in NSCLC and advanced solid tumors are summarized in Table 2. Initial results from the first-in-human phase I study for amivantamab, CHRYSALIS (NCT02609776) have recently been reported [63]. Amivantamab monotherapy was assessed in a cohort of *EGFR* exon 20 insertion mutant NSCLC after progression on platinum-based chemotherapy (median prior lines of therapy was 2) and included an efficacy population (n = 81) and a safety population (n = 114) with patients enrolled across sites from South Korea, Japan and the United States. Patients were treated with intravenous (IV) amivantamab once weekly for the first 4 weeks, and once every 2 weeks from week 5 onwards. Based on assessment of treatment with a range of 140–1,750 mg amivantamab, a maximum tolerated dose could not be identified and therefore 1,050 mg for patients < 80 kg and 1,400 mg for patients ≥ 80 kg was selected as RP2D based on available safety, pharmacokinetic and pharmacodynamic data. In the efficacy cohort (n = 81), a RR of 40% was observed including 3 confirmed complete responses (CR) and 29 partial responses (PR), while 39 patients had stable disease (SD), 8 had progressive disease (PD) and 2 patients were not evaluable. In this cohort, median PFS was 8.3 months and median OS although not yet mature is currently reported at 22.8 months. *EGFR* exon 20 insertion status was assessed by either by direct tumor sampling or circulating tumor DNA (ctDNA). In total, 25 distinct *EGFR* exon 20 insertions were identified across the cohort and importantly, PR or CR were observed following amivantamab treatment for patients who harbored insertions at all locations including in the helical region (amino acids (AA) 762–766), the near loop region (AA 767–772) and the far loop region (AA 773–775) of EGFR.

**Table 2.** Ongoing phase I and II trials involving amivantamab. EGFR: epidermal growth factor; NSCLC: non-small cell lung cancer; CNS: central nervous system; IV: intravenous; RP2D, recommended phase II dose; TBD: to be determined; RR: response rate; NR: not reported; PFS: progression-free survival; OS: overall survival; TRAE: treatment-related adverse event

Trial	CHRYSALIS NCT02609776	CHRYSALIS-2 NCT04077463	NCT04965090	PALOMA NCT04606381
Design	Phase I, open-label dose-escalation, dose-expansion	Phase I/IIb, open-label dose-escalation	Phase II, open-label	Phase Ib, open-label dose-escalation
Drug	Amivantamab	Lazertinib Amivantamab + Lazertinib	Amivantamab + Lazertinib	Amivantamab subcutaneous
Patient population	*EGFR* exon 20 insertion mutant NSCLC	*EGFR* mutant NSCLC including exon 20 insertion mutant expansion cohort B	*EGFR* mutant NSCLC with CNS metastases	Advanced solid tumors which may derive benefit from EGFR- or MET-targeted therapy
Prior lines therapy	Progression on standard of care platinum-based chemotherapy	Up to 3 lines of prior treatment in cohort B	Progression on standard of care	Progression on standard of care
Number of patients	n = 81 (efficacy population) n = 114 (safety population)	n = 520 across all cohorts	n = 40	n = 80
Dose	IV 140–1,750 mg RP2D: 1,050 mg (<80 kg) 1,400 mg (≥ 80 kg). Once weekly first 28 days, twice weekly week 5+	RP2D TBD	Amivantamab IV 1,050 mg (<80 kg) 1,400 mg (≥ 80 kg) Once weekly first 28 days, twice weekly week 5+ Lazertinib 240 mg orally once daily	RP2D TBD
RR (%)	40%	NR	NR	NR
Median PFS (months)	8.3	NR	NR	NR
OS (months)	22.8	NR	NR	NR
Dose reduction (n, %)	13% (15)	NR	NR	NR
Grade 3 or 4 TRAEs (%, (n))	35% (40)	NR	NR	NR
Reference	Park *et al*. [63]	Shu *et al*. [87]	[82]	Krebs *et al*. [88]

Notably, patients with active or untreated brain metastases were exccluded from the first phase of the CHRYSALIS study and therefore it was not possible to assess the activity of amivantamab monotherapy in patients with central nervous system (CNS) disease. Unlike small molecule inhibitors, larger mAbs often have difficulty crossing the blood-brain barrier (BBB) and are therefore typically expected to have low CNS activity [83]. Based on this rationale, a combination treatment of amivantamab with the small molecule EGFR inhibitor lazertinib is being assessed in several clinical trials (Tables 2 and 3). Lazertinib is a third-generation EGFR inhibitor which has been approved for NSCLC with T790M+ L858R or exon 19 deletion *EGFR* mutation [84]. Lazertinib has been shown to be highly CNS-penetrant [85,86] and therefore may help to compensate for an anticipated shortcoming of amivantamab to cross the BBB in patients with brain metastases. The combination of amivantamab and lazertinib versus lazertinib monotherapy will be assessed in a phase I/IIb expansion study CHRYSALIS-2 in several cohorts of NSCLC patients including cohort A: classical *EGFR* mutations with progression on osimertinib, cohort B: *EGFR* exon 20 insertions with progression on prior therapy, and cohort C: patients with rare *EGFR* mutations (e.g S768I, L861Q, G719X) [87]. CHRYSALIS-2 will exclude patients with untreated brain metastases, however, another phase II trial (NCT04965090) will specifically evaluate the combination of amivantamab and lazertinib in patients with CNS disease, split into two experimental arms of patients with parenchymal brain metastasis and patients with leptomeningeal disease with or without parenchymal brain metastasis. Lastly, the PALOMA phase Ib study (NCT04606381) will evaluate the feasibility and dosing regimen of subcutaneous administration of amivantamab in advanced solid tumors with rationale to target EGFR or MET pathways, which offers benefits in terms of a lower patient and physician burden due to reduced administration time [88].

**Table 3.** Ongoing phase III trials involving amivantamab. EGFR: epidermal growth factor; NSCLC: non-small cell lung cancer; IV: intravenous; AUC: area under curve; RR: response rate; NR: not reported

Trial	PAPILLON NCT04538664	MARIPOSA NCT04487080	MARIPOSA-2 NCT04988295
Design	Phase III, randomized first-line, open-label	Phase III, randomized first-line, open-label	Phase III, randomized first-line, open-label
Drug	Amivantamab + carboplatin-pemetrexed Carboplatin-pemetrexed	Amivantamab + Lazertinib Osimertinib Lazertinib	Amivantamab + Lazertinib + Platinum-based chemotherapy Platinum-based chemotherapy
Patient population	*EGFR* exon 20 insertion mutant NSCLC	*EGFR* exon 19 deletion or L858R NSCLC	*EGFR* exon 19 deletion or L858R NSCLC with progression on osimertinib
Prior lines therapy	None	None	Osimertinib
Number of patients	n = 300	n = 1000	n = 500
Dose	Pemetrexed IV 500 mg/m2 + Carboplatin IV AUC 5 mg/ml Day 1 of each 21-day cycle for 4 cycles Amivantamab IV once weekly, 21-day cycles. Cycle 1: 1,400 mg, Cycle 3+: 1,750 mg (<80 kg) Cycle 1: 1,750 mg, Cycle 3+: 2,100 mg (≥ 80 kg)	Amivantamab 1,050 mg (<80 kg) 1,400 mg (≥ 80 kg) 28 day cycles, once weekly in cycle 1 and every 2 weeks in subsequent cycles.	NR
Reference	[89]	Shreeve *et al*. [90]	[91]

### 4.2. Phase III

Phase III trials are ongoing in order to establish amivantamab drug combinations as first-line therapies in NSCLC, summarized in Table 3. The PAPILLON trial (NCT04538664) is currently the only phase III trial which focuses entirely on the *EGFR* exon 20 insertion mutant positive patient population. This randomized trial will assess standard of care carboplatin-pemetrexed chemotherapy treatment alone in a head-to-head comparison with a combination treatment of amivantamab and chemotherapy in the first-line setting, with PFS as the primary endpoint [89]. The following two trials, MARIPOSA and MARIPOSA-2, do not focus on *EGFR* exon 20 insertion NSCLC but are included here for context of other ongoing phase III trials evaluating amivantamab. The MARIPOSA trial (NCT04487080) is a global study across 27 countries which will compare the first-line combination of amivantamab and lazertinib against the FDA-approved treatment of osimertinib monotherapy in NSCLC patients that harbor classical *EGFR* mutations [90]. In addition, to assess the contribution of amivantamab specifically to the treatment efficacy, a third arm of lazertinib monotherapy will be included. The planned phase III trial MARIPOSA-2 (NCT04988295) will instead focus on NSCLC patients with classical *EGFR* mutations that have already progressed after osimertinib treatment [91]. In this study, patients will be randomized to either platinum-based chemotherapy alone as the control arm versus a combined treatment of amivantamab and lazertinib with platinum-based chemotherapy.

## 5. Safety of amivantamab

Amivantamab has a tolerable safety profile, with 39% of patients experiencing grade 3 or 4 TRAEs in the safety cohort (n = 114) of the CHRYSALIS phase I trial [63], which compares similarly with grade 3 or 4 TRAEs in 63% of 115 patients treated with poziotinib in the ZENITH20 trial [52] and 40% of 136 patients treated with mobocertinib in the EXCLAIM trial [56] (Table 4). Overall, the toxicity profile of amivantamab is consistent with other EGFR inhibitors, with expected toxicities resulting from WT EGFR inhibition, including rash (86% of patients), stomatitis (21%), pruritus (17%) and diarrhea (12%). In addition, amivantamab specifically has been associated with ocular toxicities, with eye disorders reported in 15 patients (13.2%) of the safety cohort from the CHRYSALIS trial, though all were reported as grade 1 or 2 adverse events. In spite of the lack of mutant over WT EGFR selectivity of amivantamab in preclinical experiments [78], the rate of severe TRAEs that led to toxicity-related dose reductions (13%) and discontinuations (10%) was relatively low. Rash was the most common cause of dose reduction (10% of patients), with discontinuation caused by rash (1.8% of patients), infusion-related reactions (IRR) (1.8% of patients) and paronychia (1% of patients). Park *et al*. suggested that the dual-targeting of MET and EGFR by amivantamab may afford additional tumor selectivity, however, the role of MET activation in *EGFR* exon 20 insertion NSCLC is poorly understood. In contrast to monotherapy with small molecule EGFR inhibitors, amivantamab treatment also leads to toxicity associated with MET inhibition. In the CHRYSALIS trial, MET-associated TRAEs included hypoalbuminemia (27% of patients) and peripheral edema (27%), although these were almost exclusively grade 1–2 events. Notably, the requirement for an IV route of administration of amivantamab results in increased patient and physician burden compared with the more common oral route of administration for EGFR tyrosine kinase inhibitors such as poziotinib and mobocertinib. Specific patient toxicities includingI IRR, which can manifest in a variety of symptoms including fever, chills, rigors among others, were frequently observed (66% of patients) following amivantamab treatment in the CHRYSALIS cohort [63]. The vast majority of IRR were associated with administration of the first treatment, with only a single IRR event reported after cycle 2. To reduce the impact of IRR, the first dose of cycle 1 was split over day 1 and day 2 of cycle 1 and prophylactic premedication was administered. Therefore, particularly for the first dose strategies to mitigate the risk of IRR are necessary and may require closer patient monitoring compared to a drug which can be administered orally.

**Table 4.** Comparison of safety profiles between amivantamab, poziotinib and mobocertinib. TRAEs: treatment-related adverse events

Drug	Amivantamab	Poziotinib	Mobocertinib
Trial	CHRYSALIS NCT02609776	ZENITH20 trial NCT03318939	EXCLAIM NCT02716116
Number of patients	114	115	136
Total Grade 3 or 4 TRAEs (%, (n))	35% (40)	63% (NR)	40% (54)
TRAEs leading to dose interruption	35% (40)	NR	54% (74)
TRAEs leading to dose reduction	13% (15)	65% (NR)	17% (23)
TRAEs leading to discontinuation	10% (11)	NR	16% (22)
Grade 3 or 4 TRAEs>5%	Hypokalemia (5%)	Rash (28%), diarrhea (26%)	Diarrhea (21%), increased lipase (5%)
Most common TRAEs (> 25%, all grades)	Rash (86%), infusion-related reaction (66%), paronchyia (45%), hypoalbuminemia (27%)	NR	Diarrhea (83%), nausea (43%), rash (33%), vomiting (26%)
Reference	Park *et al*. [63]	Le *et al*. [52]	Riely *et al*. [56]

## 6. Regulatory affairs

Based on the interim results from the CHRYSALIS phase I trial, in May 2021 the FDA granted accelerated approval for amivantamab as a treatment for NSCLC patients with *EGFR* exon 20 insertion mutations whose disease has progressed on or after platinum-based chemotherapy [45,63]. Accelerated approval for amivantamab was based on RR and duration of response and continued approval will be contingent upon verification of clinical benefit in confirmatory clinical trials [45]. As a companion diagnostic, the Guardant360 CDx liquid biopsy test was granted FDA approval alongside amivantamab to detect the presence of *EGFR* exon 20 insertions in circulating tumor DNA. Amivantamab was the first targeted therapy to be approved specifically for *EGFR* exon 20 insertions and to date, accelerated FDA approval for this patient population has only been granted to one other drug, mobocertinib, in September 2021 [44]. Currently, amivantamab remains in preregistration in the EU, Australia, Japan, Canada, Switzerland and China [92].

## 7. Conclusion

Clinical outcomes for patients with *EGFR* exon 20 insertion mutations in NSCLC have failed to improve over the last 20 years, despite significant advances for patients with classical *EGFR* mutations. Several developments including insights into the structure of the *EGFR* exon 20 insertion kinase, improvements in sequencing and detection of *EGFR* exon 20 insertions in patients and more readily available preclinical models have fueled recent drug development to tackle this unmet need. Although it was not originally designed to selectively target *EGFR* exon 20 insertions, the bispecific anti-EGFR-MET mAb antibody amivantamab has demonstrated good clinical activity against this class of mutations in the CHRYSALIS phase I trial and became the first candidate drug to be approved for this difficult-to-treat patient population. Further ongoing clinical trials will address whether amivantamab will be effective as a first-line therapy in this setting and whether outcomes can be further improved through combination treatments with chemotherapy or brain-penetrant EGFR inhibitors.

## 8. Expert opinion

From pre-clinical data and the CHRYSALIS clinical trial, it is clear that amivantamab has activity against multiple distinct *EGFR* exon 20 insertions and, coupled with a tolerable safety profile, provides a viable therapeutic option for NSCLC patients who fail to respond to standard of care chemotherapy. There remain several outstanding questions, however, and addressing the gaps in our understanding of how amivantamab works will be essential in order to move amivantamab into the front-line and design more effective treatment strategies that will deliver better patient outcomes. Firstly, the mechanistic basis that allows amivantamab to selectively target tumor cells in *EGFR* exon 20 insertion NSCLC remains unclear. There is currently no evidence to suggest *EGFR* kinase domain mutations alter the extracellular EGFR domain and indeed, amivantamab does not demonstrate good mutant over WT selectivity for EGFR exon 20 insertion mutations *in vitro* [78]. Although the biggest contribution to the therapeutic effect of amivantamab is likely due to EGFR inhibition, it is reasonable to speculate that synergistic targeting of MET must be a factor to help offer better tumor selectivity and avoid major toxicity resulting from WT EGFR inhibition. The role of MET in *EGFR* exon 20 insertion mutant driven cancers has not been investigated and only one patient enrolled in the CHRYSALIS trial was identified to have baseline *MET* amplification [63]. Preliminary evidence suggests that a high immunohistochemistry score for both EGFR and MET expression may enrich for response to amivantamab after progression on osimertinib in the classical *EGFR* mutant setting, and may represent a useful biomarker to evaluate in future studies [93]. Moreover, it is possible that dual targeting of MET will give amivantamab an advantage over other EGFR inhibitor monotherapy strategies by preventing the emergence of resistance via MET activation and thus result in a more durable response. The MET pathway is partially compensatory to EGFR signaling and MET amplification is a well-established mechanism of EGFR kinase inhibitor resistance, though whether it will be a major mechanism of resistance to inhibitors such as poziotinib and mobocertinib in *EGFR* exon 20 insertion NSCLC is not yet known [73,74].

Similarly, the mechanisms of resistance specific to amivantamab are yet to be elucidated. It is likely that owing to the fact the amivantamab binds to the extracellular domain of EGFR, the drug will be able to overcome common point mutations in the intracellular domain of *EGFR* such as T790M and C797S that are associated with kinase inhibitor resistance. In line with this, one patient on the CHRYSALIS trial with a PR to amivantamab treatment was identified to harbor a T790M resistance mutation after previous treatment with poziotinib [63]. The spectrum of resistance mechanisms that arise following amivantamab are likely to be different compared with EGFR kinase inhibitors, and can be speculated to include changes in cell surface EGFR or MET expression, mutations in other compensatory receptors including *HER2* and *PDGFRA* or pathway alterations further downstream including *RAS* mutations, such as those seen in colorectal cancers with acquired resistance to cetuximab [65,94]. Taking this into consideration, the efficacy of sequential treatment of anti-EGFR mAbs with EGFR kinase inhibitors to overcome drug resistance warrants further investigation in future studies. Similarly, chemotherapy is likely to remain effective in the short-term to extend patient survival following relapse if resistance to first-line amivantamab develops.

Lastly, it is expected that amivantamab will not cross the BBB and therefore is unlikely to have activity in NSCLC patients with brain metastases as a monotherapy. Brain metastases occur in roughly one third of all *EGFR*-mutant NSCLC patients, with comparable frequency between classical *EGFR* and *EGFR* exon 20 insertion mutant subgroups, and therefore CNS activity is an important consideration for clinical utility [42,95]. The efficacy and safety of combination strategies of amivantamab with brain-penetrant EGFR kinase inhibitors such as lazertinib remains unknown. Future and ongoing clinical trials to evaluate these combinations will therefore be an important hurdle for amivantamab to cross and will inform whether first-line strategies can achieve long-term benefits in NSCLC patients with and without brain metastases.

## Article highlights

- NSCLC patients harboring *EGFR* exon 20 insertion mutations face poor outcomes to first-, second- and third-generation EGFR kinase inhibitors
- The bispecific anti-EGFR-MET monoclonal antibody amivantamab demonstrates clinical activity against *EGFR* exon 20 insertion mutations
- Based on the CHRYSALIS phase I trial, amivantamab was approved in May 2021 by the FDA for *EGFR* exon 20 insertion positive NSCLC after progression on chemotherapy
- The amivantamab antibody molecule is not selective for EGFR exon 20 insertions yet demonstrates a tolerable safety profile in patients, suggesting a potential hypothesis that dual MET inhibition enhances tumour selectivity
- Amivantamab is unlikely to cross the blood-brain barrier, and therefore its clinical utility as a monotherapy may be limited in patients with brain metastases
- Phase III trials that will evaluate amivantamab in combination with chemotherapy or brain-penetrant EGFR kinase inhibitors will generate important data to determine its efficacy in the first-line setting
